# Supplementary figures and images for: Modulation of ACD6 dependent hyperimmunity by natural alleles of an Arabidopsis thaliana NLR resistance gene
Source: PLoS Genet. 2018 Sep 20;14(9):e1007628. doi: 10.1371/journal.pgen.1007628 (PMC6168153; doi:10.1371/journal.pgen.1007628)

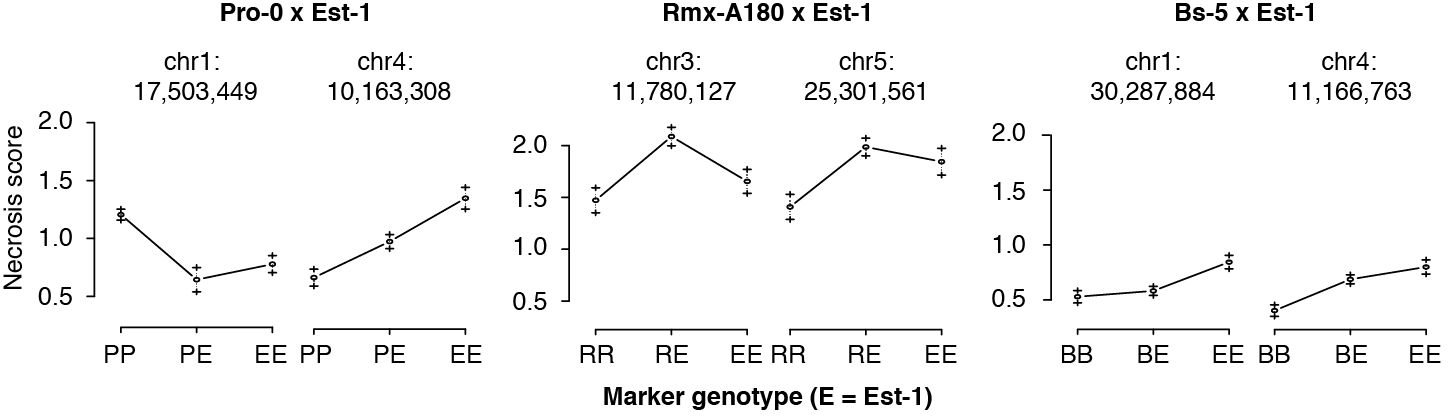

Supplement: S1 Fig — (TIF) [file pgen.1007628.s001.tif]

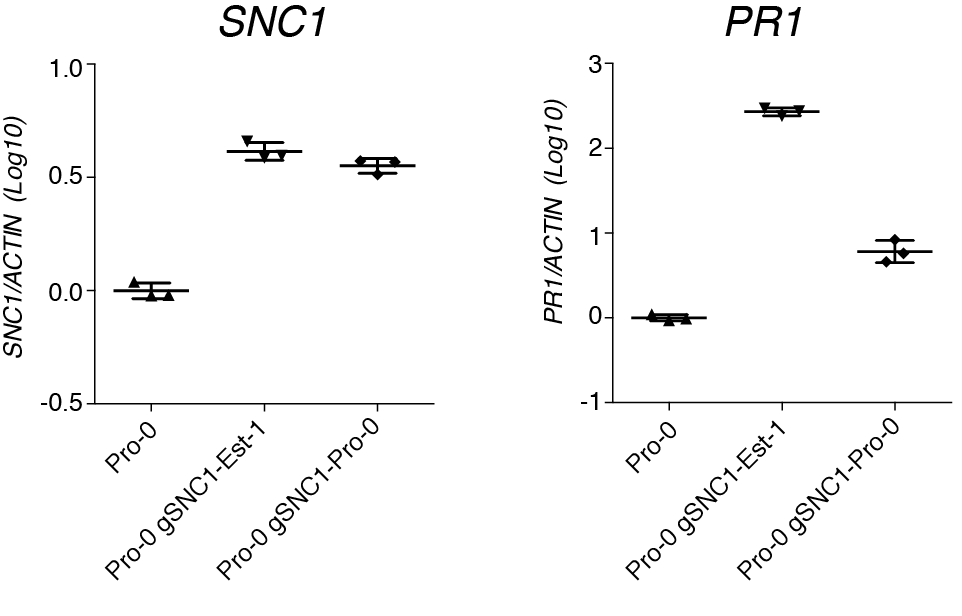

Supplement: S2 Fig — (TIF) [file pgen.1007628.s002.tif]

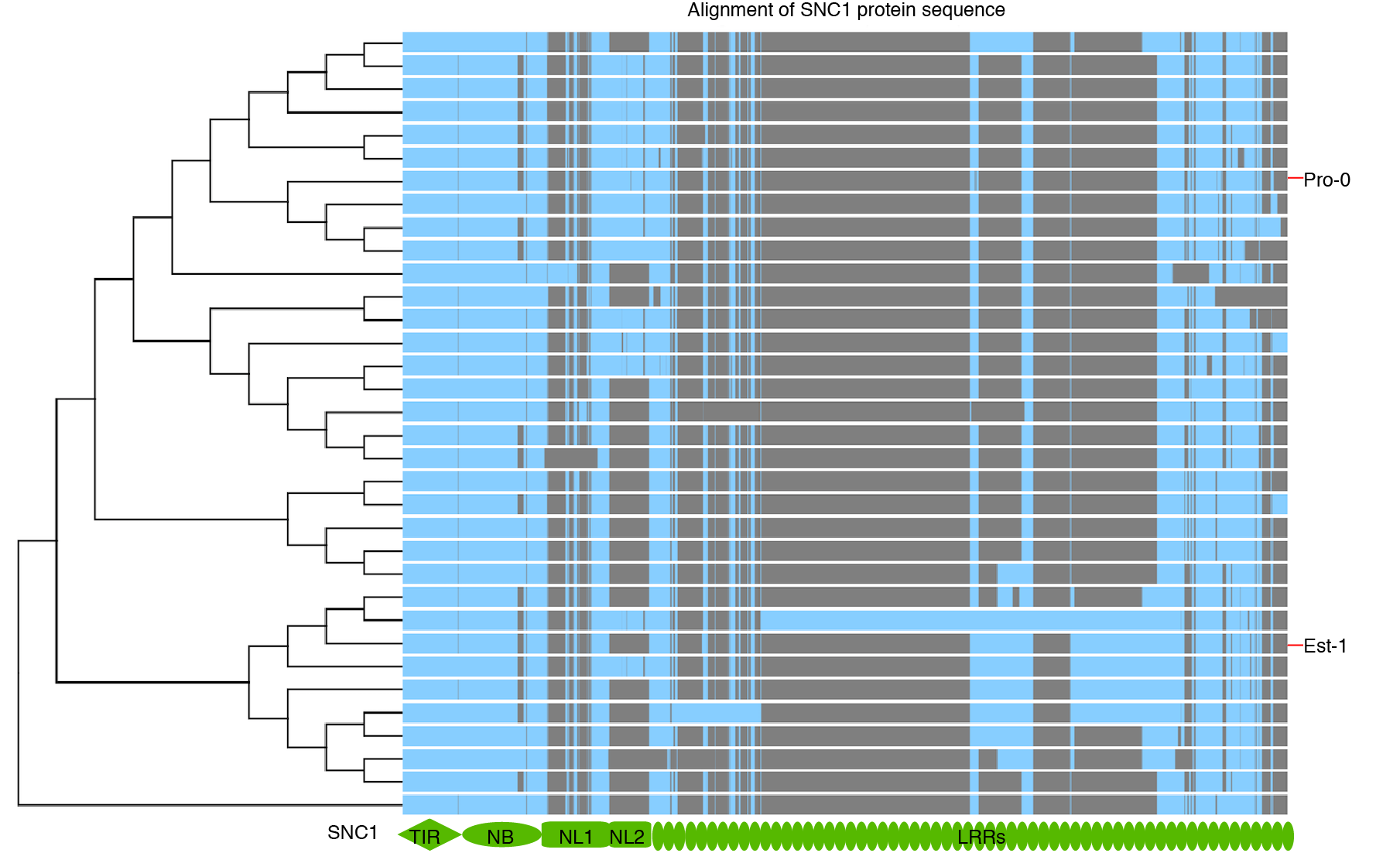

Supplement: S3 Fig — Gray indicates structural variants including small and large deletions. (TIF) [file pgen.1007628.s003.tif]

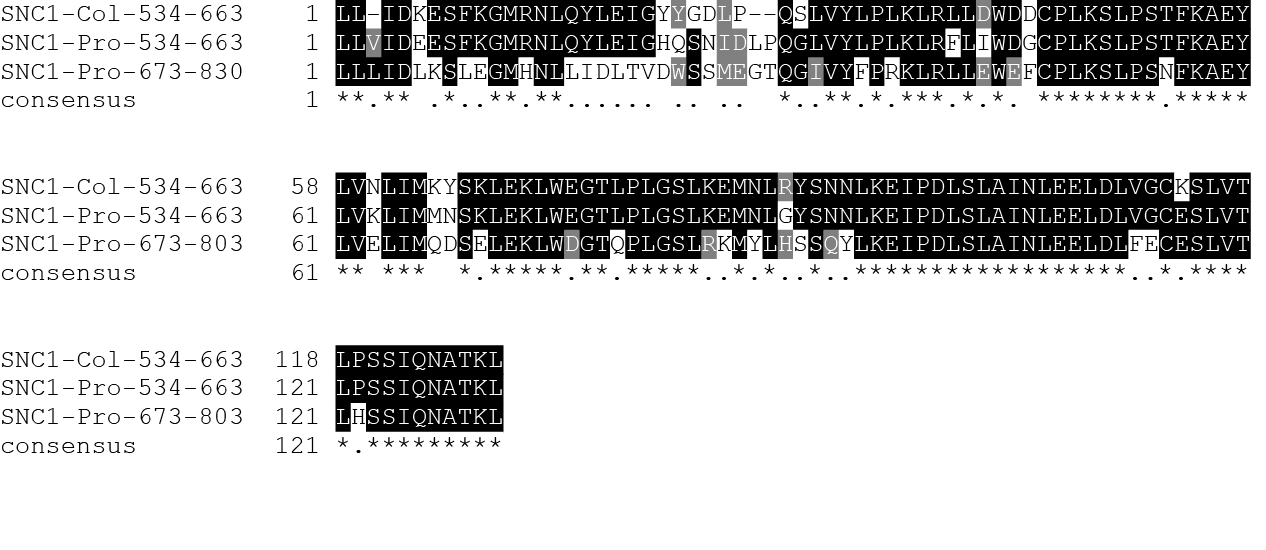

Supplement: S4 Fig — (TIF) [file pgen.1007628.s004.tif]

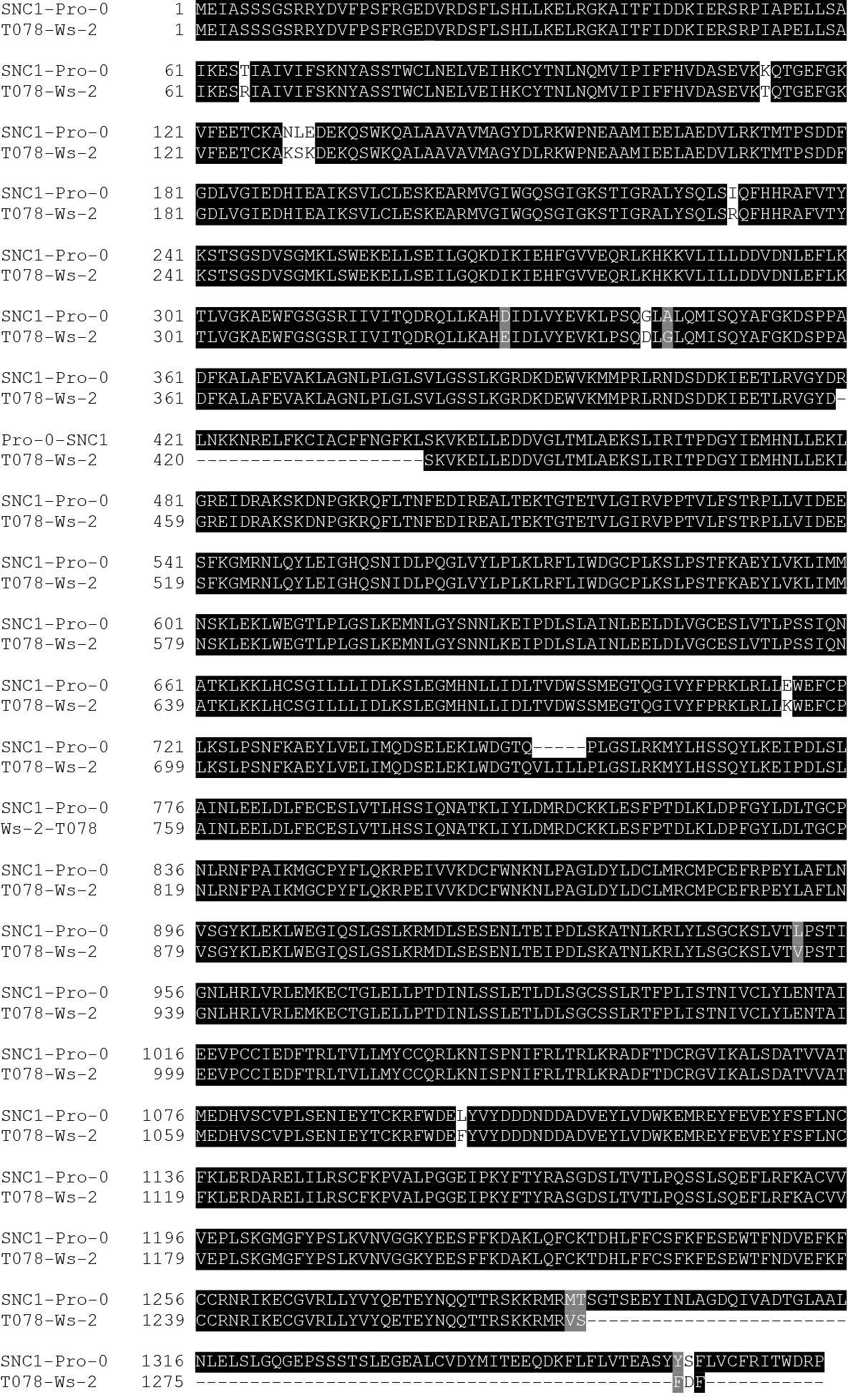

Supplement: S5 Fig — (TIF) [file pgen.1007628.s005.tif]

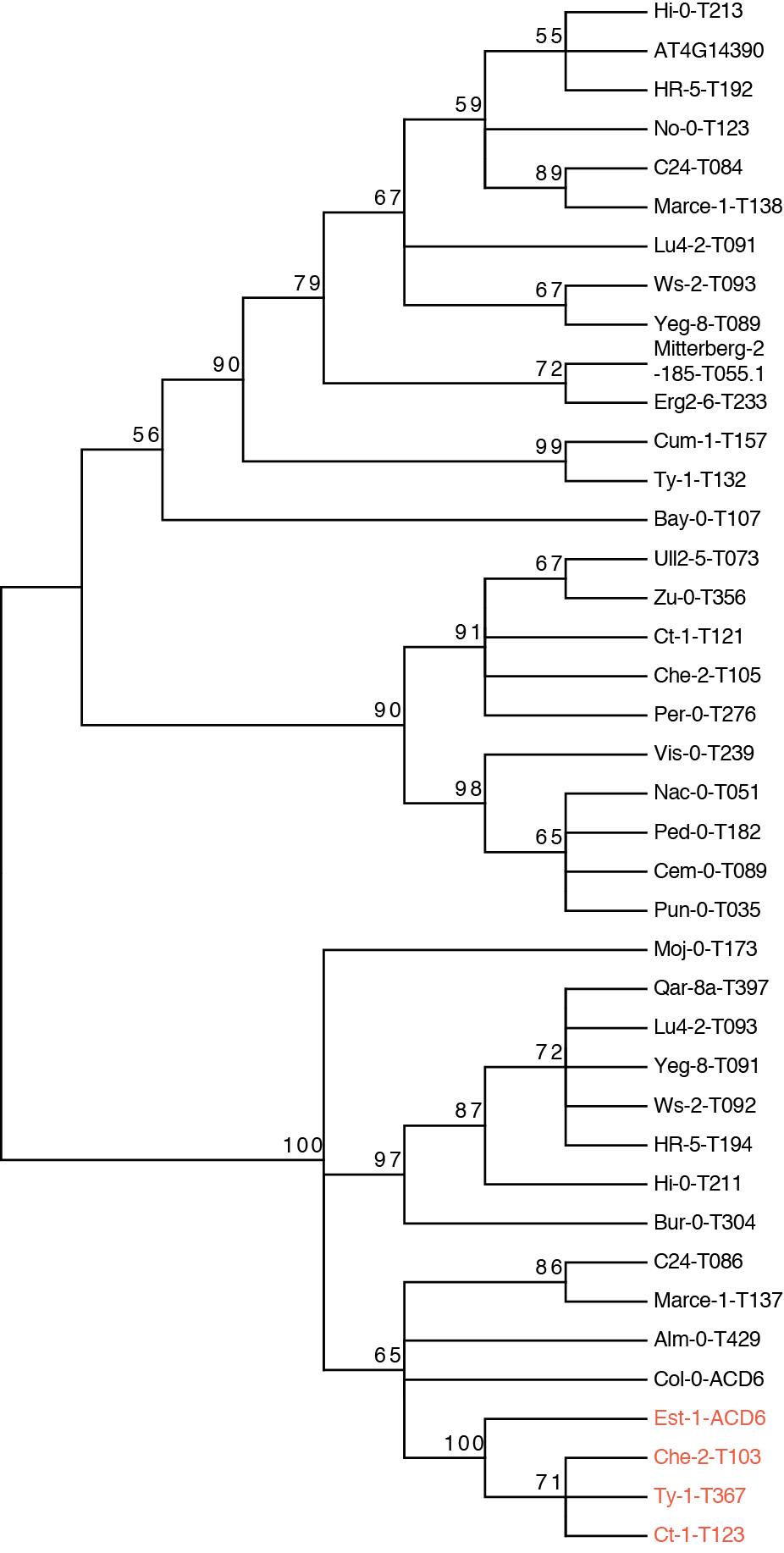

Supplement: S6 Fig — Bootstrap values over 60% are indicated. Est-1-like clade highlighted in red. (TIF) [file pgen.1007628.s006.tif]

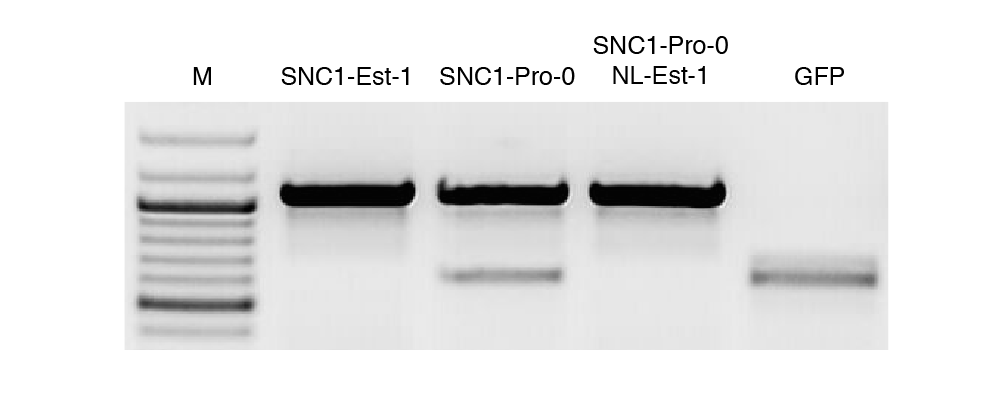

Supplement: S7 Fig — Samples were harvested 2 d after infiltration. (TIF) [file pgen.1007628.s007.tif]

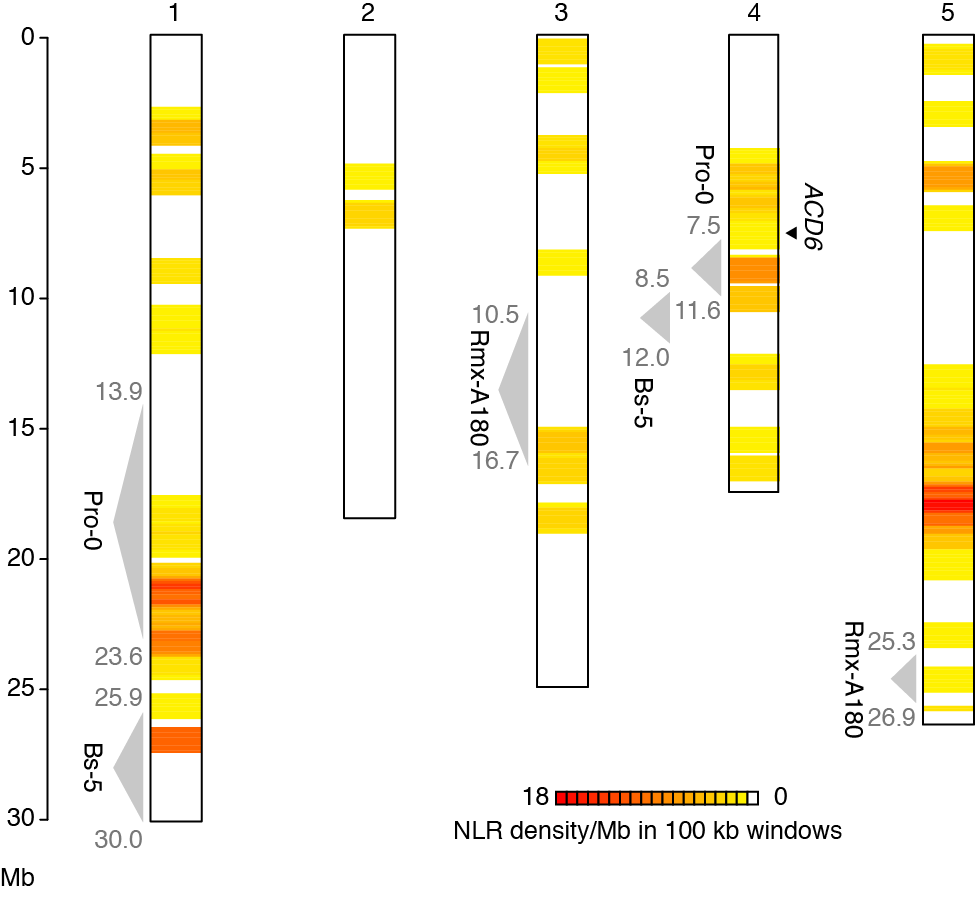

Supplement: S8 Fig — QTL intervals are indicated by unfilled triangles. (TIF) [file pgen.1007628.s008.tif]
